# Biodesign program introduction in Japan: promotion of entrepreneurship and viewpoints of education on medical technology innovation

**DOI:** 10.1007/s10047-022-01317-4

**Published:** 2022-03-03

**Authors:** Koji Nakao, Mitsuo Umezu, Kiyotaka Iwasaki

**Affiliations:** 1 Cooperative Major in Advanced Biomedical Sciences, Joint Graduate School of Tokyo Women’s Medical University and Waseda University, Waseda University, 2-2 Wakamatsucho, Shinjuku, Tokyo, 162-8480 Japan; 2 Institute for Medical Regulatory Science, Waseda University, Tokyo, Japan; 3 Department of Integrative Bioscience and Biomedical Engineering, Graduate School of Advanced Science and Engineering, Waseda University, Tokyo, Japan; 4 Department of Modern Mechanical Engineering, School of Creative Science and Engineering, Waseda University, Tokyo, Japan

**Keywords:** Biodesign, Entrepreneurship, Innovation, Education, Medical technology

## Abstract

The Stanford Biodesign program was first introduced in Japan in 2015 at three national universities to develop medical technology innovation and its talent. This study aimed to (1) show the outcomes of leadership talent development, (2) indicate the educational results of the program, and (3) objectively analyze the ways in which the program executed in Japan, effectively promoted entrepreneurship orientation and the origination of new businesses. The latter is especially relevant as Japan has low entrepreneurial awareness and new business entry rates compared to the United States and Europe. Herein, fellows were subjected to questionnaires, interviews, and a survey based on academic papers, extant literature, and treatises issued by the Nihon Biodesign Gakkai (Academic Society of Japan Biodesign). Overall program performance showed notable results, despite indicating a need to improve business-related programs and team learning which is greatly influenced by Japanese culture. An externship program, planned and developed in Japan, was most inspiring and served to expose participants to role models. Comparing Japan Biodesign education elements to factors of general entrepreneurship promotion in Japan, sampled and organized from relevant White Papers, proved its educational effectiveness in entrepreneurship promotion from an objective viewpoint. Within the 4-year timeframe, the results indicated that leadership talent was indeed developed. Medical device innovation should progress through the stages of establishing new ventures, followed by contriving medical devices with novel, impactful value. This study revealed that Japan Biodesign education provides a platform for achieving these goals, despite the challenging Japanese new business environment.

## Introduction

In 2015, the Stanford Biodesign program [1, 2] was first introduced to three national universities in Japan (Tohoku University, University of Tokyo, and Osaka University) with the support of the Japan Federation of Medical Devices Associations, the universities, and governmental authorities. The rationale behind this introduction was to further promote medical device innovation and develop leadership talent, as well as contribute to global healthcare by expanding the domestic medical device industry. The Stanford Biodesign education program has been adopted at many universities within and outside of the United States (US) and has yielded several papers [3–6].

A unique feature of the Biodesign program is its “Needs Start” innovation process that comprises the major steps, “Identify,” “Invent,” and “Implement” and starts with finding and screening unmet medical needs. Overall, the education offered in this curriculum consists of three elements, namely, medical device and business-related programs, and a design thinking approach, which supports the processes of the former two. Compared to the US and Europe, entrepreneurial awareness and activity are quite low in Japan, and very few new ventures arise. Thus, externships were established for, and incorporated into, the Japan Biodesign education program.

This study aimed to analyze the degree to which Biodesign education in Japan has contributed to developing leadership talent and determine how to rate the overall education program given the remaining issues. Moreover, its effectiveness in spurring on an entrepreneurial orientation that could lead to new ventures and innovation was evaluated, and the type of factual results produced across the 4-year long terms—from program inception in 2015–2019—are determined. The outcomes of this study provide suggestions for directing medical device innovation education in Japan.

## Materials and methods

### Development of leadership talent

A main objective of Biodesign education is the development of leadership talent. In 2015, the Japan Biodesign Program was introduced to three universities, Tohoku University, University of Tokyo, and Osaka University. The results of a questionnaire survey conducted in 2019 by the Nihon Biodesign Gakkai (Academic Society of Japan Biodesign) [7] on 41 fellows in the Japan Biodesign Program between 2015 and 2019, and the results of interviews were used and analyzed to determine the extent to which leadership development had occurred. The secondary use of the questionnaire results has been approved by the survey participants at the time of the questionnaire survey. This study was approved by the Ethics Review Committee on Research with Human Subjects of Waseda University (2021-337).

Leaders were defined as people who: (1) decided to start new businesses upon consideration of personal risks, (2) assumed project leader positions because of recognition of their Biodesign experience and took charge of planning, and (3) rose to positions closer to the CEO within organizational layers (promotion was excluded). The research period extended from completion of the program through March 2021. Forty-one individuals were interviewed by phone between April and June 2020, and their responses were analyzed according to the stated definition.

### Overall program rating

A questionnaire-based survey was conducted from August to September 2019; the effective response rate was 100%, and responses were sent to the faculty of Nihon Biodesign Gakkai. In total, 41 fellows from the four terms included in this study responded and evaluated each program subject. A 5-point scale was used to evaluate the main subjects using question responses: strongly agree = 5, somewhat agree = 4, agree = 3, somewhat disagree = 2, and disagree = 1.

One of the evaluation subjects was the Team Approach, which is an important factor in design thinking. A team with maximum four members was formed at each university. Where possible, effort was made to select group members with diversity in mind, combining individuals from medicine, engineering, and business sectors, and of both sexes. However, certain challenges arose, including that fewer fellows were available than anticipated, and the number of women was overwhelmingly small (7/41 team members overall, 17.1%). Since medical device development requires knowledge of both medicine and engineering, there was a need for at least one team member to be from the medical and engineering field. Over the 4-year period, 12 teams (3 universities × 4 years) were formed, and fortunately, members with medicine and engineering backgrounds joined each team. The degree to which the fellows recognized and understood the importance of the team approach to the overall program was evaluated.

To promote team performance, a Team Learning program was introduced from the second term onward. A specialist came onboard and started mentoring the teams by providing lectures and guidance four times a year. The concept was to ensure the psychological safety of members so they could function as a team [8]. The key to team learning was for team members to determine how to communicate with each other (i.e., expressing words, attitudes, feelings, and mutual understanding). Team learning aimed to answer the ultimate question: “Which is more important, harmony with others or speaking frankly?” Since achieving the team objective (medical device innovation) takes priority, diverse and meaningful ideas are most important. Coaching and mentoring were geared toward encouraging such communication. A questionnaire-based survey was conducted that required fellows to score the effectiveness of team learning; we anticipated some difficulties in this regard because frank communication and psychological safety are highly associated with Japanese culture.

An externship program was incorporated into the Japan Biodesign education curriculum. The program entailed sending fellows to ventures or incubators in Silicon Valley for 2–4 weeks to gain hands-on experience while learning in an actual medical device start-up setting. This was done against the backdrop of Japan’s low level of entrepreneurial awareness and limited number of new ventures, compared to the US and Europe. Naturally, this translates into a low absolute number of ventures in Japan, whether in the medical device field or otherwise. Accordingly, a rating system that listed nine items—including externships—was used, allowing fellows to choose their three most preferred programs, to examine the results.

### Entrepreneurial orientation

A questionnaire-based survey was conducted, to which 41 fellows responded. Then, changes in the entrepreneurial orientation of fellows before enrollment and after completion of the program, were examined.

Using the 2011 [9], 2014 [10], and 2017 [11] White Papers on Small and Medium Enterprises in Japan, Venture White Paper 2019 [12], and other materials [13–15], the general state of entrepreneurship, including entrepreneurial awareness and the factors of entrepreneurial orientation promotion, were researched and examined. Then, general entrepreneurial orientation factors were compared to relevant program elements to objectively verify the degree to which the Japan Biodesign program effectively promoted entrepreneurship. There are specific reasons for adopting the abovementioned source documents are. On the one hand, the White Papers on Small and Medium Enterprises in Japan are issued by the Small and Medium Enterprise Agency of the Ministry of Economy, Trade and Industry, which is a government agency. These reports reflect entrepreneurship data surveyed at regular intervals, and all mentioned editions survey entrepreneurial awareness and entrepreneurship promotion factors. These institutional data, therefore, offer reliability and consistency, which are key factors in this research. The Venture White Paper report has been issued annually since 2012 by a body—currently known as the Venture Enterprise Center—established with the support of the Ministry of International Trade and Industry in 1975. They conduct an ongoing survey of the state of entrepreneurship and entrepreneurship awareness, and resultantly, their report is considered a consistent and reliable source of data. Moreover, these White Papers are often cited in the literature.

For a meaningful comparison, the factors that promote entrepreneurship were examined and chosen as follows: all factors related to “motive, purpose, and opportunity” for promoting entrepreneurship stated in these White Papers were listed, followed by the exclusion of factors unrelated to Biodesign education. For example, “self-realization” depends on an individual’s personal thoughts or determination, and “personal discretion” is excluded because starting one’s own business requires using personal discretion. As shown in Table 1, excluded factors are grouped under “(6) Others,” whereas the remainder are listed as factors (1) through (5). These general entrepreneurship promotion factors and relevant elements from the Biodesign program were compared, and the comparison was used to evaluate the questionnaire results to enable an objective evaluation of how the overall program promoted entrepreneurial orientation.

**Table 1:** Comprehensive list of “motive, purpose, and opportunity” factors

Relevant promotion factors (excluded factors are listed under “Others”)
Contribute to society
Use specialized skills and knowledge (including hobbies and special skills)
Commercialize ideas
Role models (influential entrepreneurs, acquaintances of entrepreneur, e.g., friends, seniors, etc., famous successful entrepreneurs)
Friends and associates who have the same desire and/or may become business partners
Other Self-realization; personal discretion (including ability to do what one wants in the workplace); anxiety about the future and worsening treatment in the workplace; recommended by others (family, friends, business partners, etc.); encouraged by parents, school teachers, company bosses, colleagues, or relatives other than parents; desire for higher income; desire for social status as a manager; ability to work regardless of age or sex; for time and mental space; ability to work flexibly while handling housework, parenting or caring for elderly family members; influenced by parents or relatives; accustomed to entrepreneurship from a young age; retired; no other work openings available; request from parent company, etc.; desire to make effective use of real estate and other assets; stories and speeches (at school, seminars, television, interview, online)

### Entrepreneurship

Factual results, such as the number of start-ups and patent applications, subsidies, and venture capital (VC) investment, were surveyed by interviewing all fellows from August 2019 to March 2021.

## Results

### Development of leadership talent

**Fig. 1:**
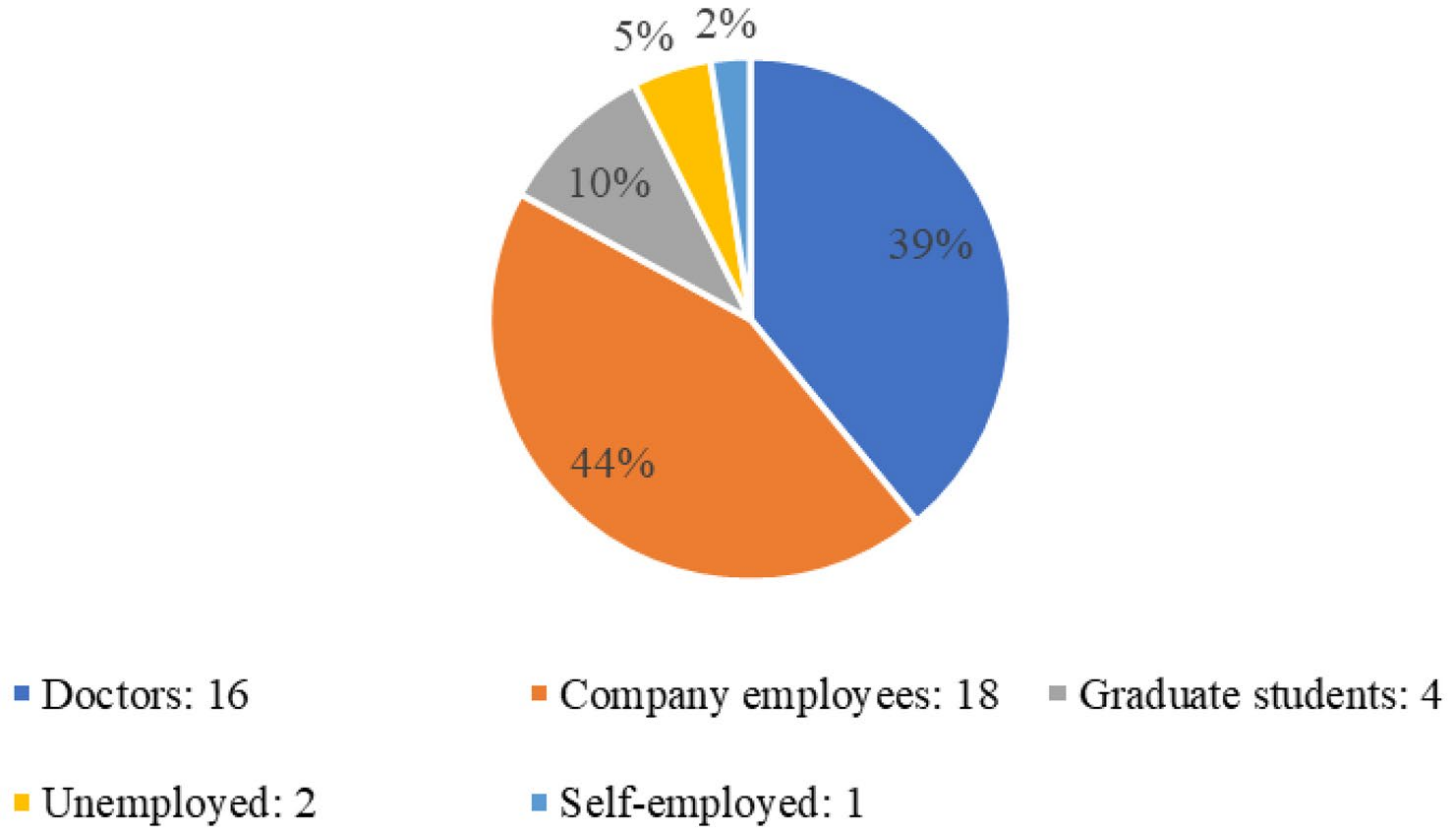
Breakdown of fellows (41 members)

Originally, 48 places were available, and 42 individuals accepted into the program, of which 41 ultimately completed it (85.4% fulfillment rate = 41 fellows/48 places). The mean age of participants was 33.0 years, and the male/female ratio, 83:17. Figure 1 shows the career breakdown of all 41 members; company employees and doctors (18 and 16 individuals, respectively) accounted for 82.9% of participants, followed by graduate students, as well as unemployed and self-employed individuals.

Table 2 shows the results of the leadership talent survey. Leadership talent was observed in 25 participants (61.0%).

**Table 2:** Leadership talent per career

Career	Entrepreneurship	Leadership position	None	Total	Leadership talent ratio
Doctors	10*	3*	4	16*	75.0
Company employees	0	10	8	18	55.6
Graduate students	1	0	3	4	25.0
Self-employed	1	0	0	1	100.0
Unemployed	0	1	1	2	50.0
Total	12	13*	16	41	61.0%

*One doctor started a new venture and at the same time, he was appointed as a project leader. In the total section, it was counted as one

### Overall program rating

The questionnaire items shown in Table 3 were surveyed on a 5-point scale as follows: strongly agree = 5, agree = 4, neutral = 3, somewhat disagree = 2, disagree = 1. The response rate was 100%.

**Table 3:** Questionnaire results (in order of questions)

Question items	Average score	95% CI	Total of “strongly agree” and “agree”
Were you satisfied with enrollment in Biodesign?	4.80	4.68, 4.93	100.0%
Would you continue the program if possible?	3.63	3.26, 4.01	58.6
Would you recommend the program to others?	4.22	3.93, 4.51	80.4
Did you learn the process of Needs Start?	4.71	4.55, 4.87	97.6
Did you learn about business?	3.73	3.47, 4.00	75.6
Did you learn team learning?	4.17	3.91, 4.43	85.4
Did you learn design thinking?	4.36	4.16, 4.57	95.1

In evaluating the overall program, Question 1 (“Were you satisfied with enrollment of Biodesign?”) scored 4.80 on average, and follow-up Question 3 (“Would you recommend the program to others?”), received a high average score of 4.22. Question 2 (“Would you continue with the program if possible?”) may have been meaningless for graduates who had already left the program two or more years earlier. Question 4 (“Did you learn the process of Needs Start?”), which relates to a feature of design thinking, scored 4.71, whereas Questions 6 (“Did you learn team learning?”) and 7 (“Did you learn design thinking?”) scored 4.17 and 4.36, respectively, which were all high average scores. However, Question 5 (“Did you learn about business?”) scored a relatively low score of 3.73. The proportion of participants who responded with “strongly agree” and “agree” is presented in the right-hand column; this did not differ notably from the average value on the 5-point scale, suggesting a need for improvement in education related to business.

The variable, “Significance of team approach”, achieved a score of 4.78, at an effective response rate of 100% (Table 4), indicating that the fellows clearly grasped its importance. In evaluating the results of the team learning program, the key question was “Which is more important, harmony with others or speaking frankly?” (psychological safety, importance of effective ideas); although fellows allocated a high score of 4.48 to “Was mentoring support useful?”, the average score dropped to 4.12 in response to “Did your own attitude and speech change?” and 4.00 in answering “Did your team become more effective?” when they were asked to provide more in-depth responses. Furthermore, in response to the question, “Were you able to speak frankly to faculty and team fellows towards the end of the total program?” 61.3% indicated that they indeed found it difficult. However, even though fellows who answered “Yes” were asked to elaborate on the biggest factor hindering them to speak frankly, 42.1% of them did not indicate the factor at all. Although difficulties were anticipated from the outset, it is nonetheless disappointing that the team learning program apparently requires improvement, such as an increase in the number of lectures.

**Table 4:** Significance of team approach and effects of team learning

Survey subjects	Question	Average score	95% CI	Effective response rate (%)
Significance of team approach				
Phases 1–4, 41 persons	Was team building significant to the program overall?	4.78	4.60, 4.96	100
Effects of team learning				
Phases 2–4, 31 persons	Did mentoring and support by a specialist and faculties make team approach more effective?	4.48	4.19, 4.78	100
	Did your own attitude and speech change as a result of mentoring and support?	4.12	3.87, 4.39	100
	Was your team more effective as a result of mentoring and support?	4.00	3.67, 4.33	100

Table 5 shows answers in response to a request to “Choose the best three programs you have experienced”. “Externship” received the highest score from 30 fellows, making it the most frequent choice. Based on the comments—excluding those from one person who visited a venture when only one employee was present, due to bad timing—everyone learned a great deal and found the experience very inspirational, clearly inferring the allocation of high scores. This is supported not only by the number of respondents but also by the comments themselves. The second most frequently selected option—from 26 respondents—was “Established a network.” The selection frequency of these two variables indicated the factors that impacted entrepreneurial orientation promotion, the most.

**Table 5:** Number of respondents for each item

Number of respondents	Items
30	Externship (visit to Silicon Valley)
26	Established a network (fellows, alumni, faculty, outside lecturers)
23	Needs identification
17	Clinical immersion
10	Intellectual property (IP), regulatory approval, insurance reimbursement
6	Commercialization (development strategy, plan design, project planning)
5	Team learning
5	Bootcamp
3	Creating and making pitches and presentations
2	Concept creation

Total of 127 responses (3.1 responses per person) with a 100.0% effective response rate. Although it was assumed that one person would give a maximum of three answers, two gave six, and two gave two. The average number of responses was 3.1, so the results were used as they were

### Entrepreneurial orientation

**Fig. 2:**
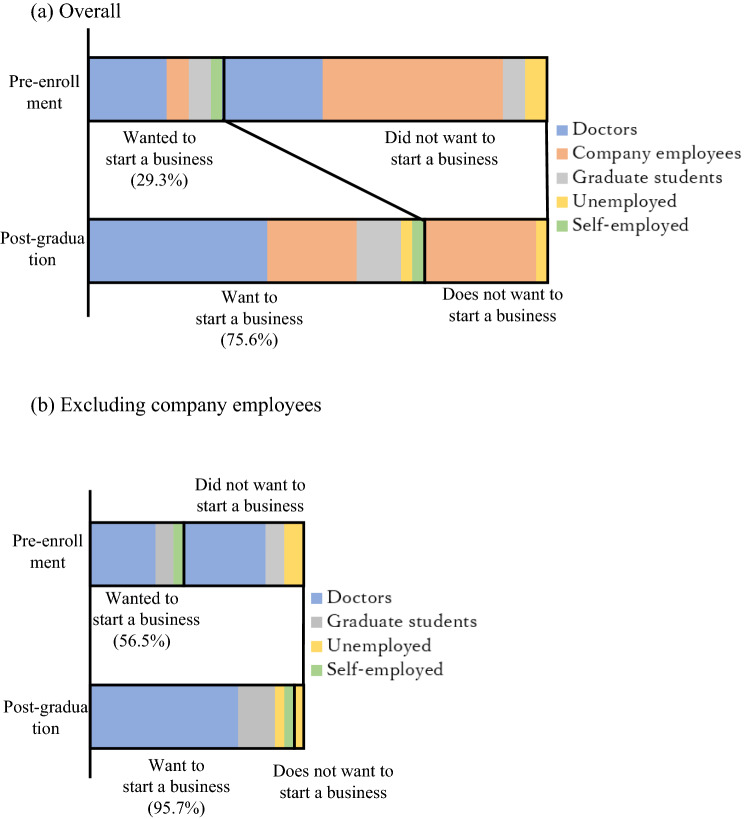
Entrepreneurial orientation

Changes in the fellows’ entrepreneurial orientation before and after the program were investigated by analyzing results from two groups; the first included all fellows, and the second, all except company employees. The latter grouping was based on the assumption that few company employees would leave their employment and start a business immediately after finishing the program due to company support in form of a stable income and job security. As expected, during the survey period, there were no cases of employees leaving their companies and starting businesses after completing the Japan Biodesign program. Twelve people (29.3%) answered “Yes” when asked whether they “Wanted to start a business” before enrollment. Of these, 43.8% were doctors, compared to only 17.0% company employees (Fig. 2). Conversely, all doctors said they “Wanted to start a business” upon program completion, which was the case for only half of them, before enrollment. Moreover, company employees with entrepreneurial aspirations increased from two to eight people, all four graduate students were inclined to entrepreneurship, and one of the two unemployed individuals switched to an entrepreneurial orientation during the course. Overall, 31 of 41 participants (75.6%) changed their inclination to “Wanted to start a business,” and 18 of the 29 who initially did not want to start a business switched to an entrepreneurial orientation (65.5%). When company employees were excluded, only one unemployed person “Did not want to start a business,” whereas the remainder of respondents wanted to start a business, amounting to a rate of 95.7%.

We further compared general entrepreneurial orientation factors (Table 1) and programs, examining the degree to which the Biodesign program objectively affected the promotion of entrepreneurial orientation.

The variable, “Contribute to society,” may be regarded as contributing to healthcare in the case of medical device development. In total, 2483 needs were identified through clinical immersion and reduced to a final 15 based on relevant medical and commercial criteria and impact; the identification of needs was based on describing the best way to provide medical results and to whom. Teams chose and proposed an engineering solution (technological possibilities) based on the study of the literature, relevant hypotheses, and intellectual property (IP) possibilities while building simple prototypes. These activities encompassed the “Use of specialized skills and knowledge (including hobbies and special skills)” and “Commercialization of ideas.” Business plans were formulated covering research and development (R&D), proof of concept, regulatory path, distribution, and marketing, whereafter commercial possibilities were explored. Overall, the program addressed the factors of “Contribute to society (medical contribution),” “Use specialized skills and knowledge,” and “Commercialize one’s ideas.”

Concerning “role models” (“Externship which provides fellows with direct contact with and exposure to real ventures and incubators”) and “Friends and associates who have the same mind and may become business partners,” the questionnaire results related to “Externship” are addressed first. Table 5 shows the results of fellows choosing their three most preferred programs. “Externship” was selected most often (30 respondents), which supports that fellows had the greatest impression of and were most inspired by this program element, and it clearly served to provide exposure to role models.

Next, we studied “Friends and associates of the same desire and of the potential business partners” Teams worked together throughout all processes, resulting in development of a network between team members, alumni, faculties, and lecturers, which later proved to be a valuable asset. This corresponds to the second most preferred answer in Table 5, “Established a network,” which was scored by 26 respondents.

A comparison between general factors for entrepreneurship promotion and the Japan Biodesign program is shown in Table 6, indicating that the program elements clearly corresponded to each objective factor.

**Table 6:** Comparison of general entrepreneurship promotion factors and Japan Biodesign

General entrepreneurship promotion factors	Corresponding the Japan Biodesign program (including externships)
Contribute to society	Medical device innovation contributes to healthcare and society, which is the goal of Biodesign
Utilize specialized skills and knowledge	Proposing medical device innovation requires utilization of skills and knowledge at both the personal level and by team members
Commercialize ideas	Program provides opportunities to learn business-related subjects
Role models	Externships provide inspiration through direct contact with actual ventures, that is, role models
Friends and associates with the same business-related desires and potential business partners	Biodesign requires all members to go through all processes as a team, thus creating valuable network with fellows, alumni, faculties, and lecturers

### Entrepreneurship

Table 7 shows that entrepreneurs constituted 29.3% of all fellows, or 52.2% of participants, excluding company employees.

**Table 7:** Entrepreneurship results

Item	Total
Start-ups Entrepreneurs (overall) (excluding company employees)	6 companies 12/41 persons (29.3%) 12/23 persons (52.2%)
Subsidies	235.5 million yen
Venture capital investment	413 million yen
Number of patent applications	14

All data were based on the results before the end of March 2021

## Discussion

### Development of leadership talent

A reasonable number of fellows were recruited over the 4-year period, with an overall fulfillment rate of 85.4%. As the name “Biodesign” was unfortunately not known in the early years of this program being presented in Japan, recruitment efforts were needed to continually make potential fellows aware of the program. An increase in applicants would result in a positive cycle, enabling us to attract higher talent with passion, which would result in successful innovation and leader development. The recruiting activity is a major issue in Biodesign education in Japan. As for leadership talent, it may be important to periodically verify the status over a certain period.

### Overall program rating

Based on the questionnaire results, the overall program was highly rated by the fellows. As shown in Table 3, the average scores for both “Were you satisfied with enrollment in Biodesign?” and “Would you recommend the program to others?” were high (4.80 and 4.22, respectively), as was the total proportion of “strongly agree” and “agree” responses (100.0% and 80.4%, respectively). While the significance of the team approach was well understood, questionnaire results suggested that improvement is needed in team learning, which is deeply associated with Japanese social hierarchies and culture. It was implied that frequency of mentoring and support should be increased—presently, contact sessions are held four times per year. The question, “Did you learn about business?”, scored a low 3.73, indicating that improvement is needed. The program was already changed to teach business class with more resources (contents and time). Furthermore, post graduate education was created. When fellows have questions or face real problems, they have an opportunity of mentoring and consultation from business specialists for real solutions. “Externships” (role models) and “Established a network” (with friends and associates) both showed excellent results.

### Entrepreneurial orientation

Major changes were observed in the fellows’ entrepreneurial orientation before enrollment and upon course completion, indicating that the program was very effective in this respect. Prior to enrollment, 12 of 41 people (29.3%) wanted to start a business, and the number changed to 30 individuals (73.2%) after completion of the program. When excluding company employees, the number of participants who wanted to start a business increased by 52.2% from 10 (43.5%) pre-enrollment to 22 (95.7%) post-graduation. Thus, most of the fellows in this category developed a stronger entrepreneurial orientation.

To better understand the situation regarding entrepreneurship and new ventures in Japan, we investigated some statistics. As described by an international comparison in the 2017 White Paper on Small and Medium Enterprises in Japan [6], the new business entry rate remained between 4.4% and 5.2% in Japan, while it hovered between 10.4% and 9.3% in the US, for the period from 2001 to 2015. Furthermore, the lack of interest in entrepreneurship (from 2001 to 2012) differed substantially between the two countries, with the data indicating the rage between 30.0 and 22.9% in the US, compared to 75.8–77.3% in Japan. The level of entrepreneurship (people think of more opportunities in new businesses) was 7.0% in the US, compared to 3.0% in Japan. Moreover, the percentage of the adult population that was hesitant about entrepreneurship due to strong fears about business failure, was 49.0% in Japan; the fourth highest level in the world. The proportion of the adult population that believed there were good opportunities for entrepreneurship, was 7.0% in Japan, which differs greatly from the 51.0% and 41.0% in the US and United Kingdom, respectively. Reasons for the low level of interest in entrepreneurship in Japan, included “no opportunities to come into contact with entrepreneurs due to lack of acquaintances,” “strong inclination towards life or economic stability,” and “no room to take risk such as failure” [13–15]. No statistical data were available to evaluate the number of ventures in the medical device field specifically, but similar to the overall picture, few medical device ventures exist in Japan. Against this backdrop, the externship education programs of the Japan Biodesign initiative scored the most points after 30 respondents selected is as their most preferred experience. Judging from fellows’ comments, direct contact with and exposure to role models inspired them that “seeing is believing.”

As shown in Table 6, each Biodesign program element corresponded with a general entrepreneurial orientation factor, indicating that the program was objectively effective in promoting entrepreneurial orientation. The findings indicated a valuable fit between the original Biodesign program and the externship program implemented in Japan. Becoming an entrepreneur or launching a new venture is a personal decision involving an individual’s career, passions, risk tolerance, financial situation, life stage, and so on.

**Fig. 3:**
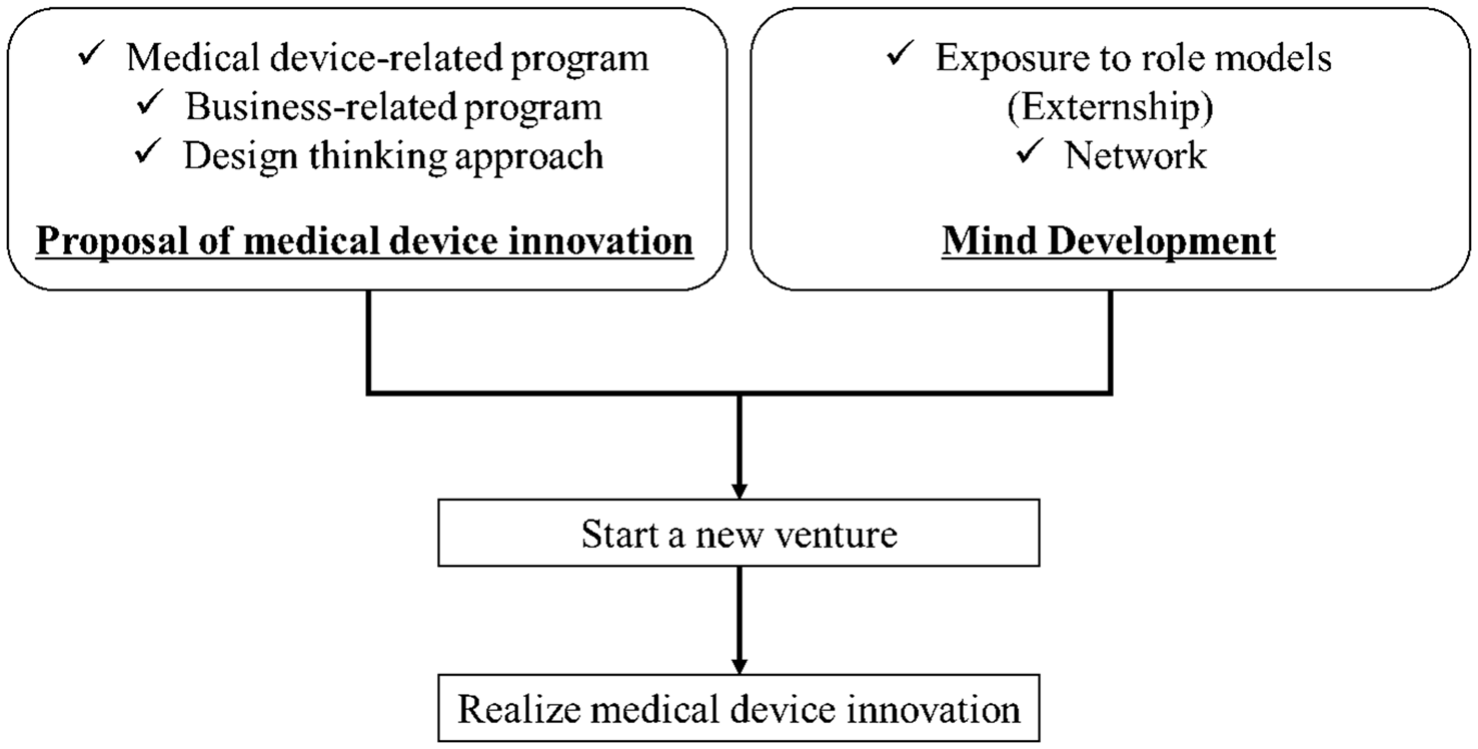
Steps to medical device innovation

As shown in Fig. 3, the Japan Biodesign education program is a practical study that enables fellows to learn about medical devices, business, and a design thinking approach that culminate in proposing device innovation [16], while they have important opportunities to see real ventures in action and establish networks. Japan Biodesign provides an important platform for reaching the next steps, which are to set up a new venture and realize innovation.

### Entrepreneurship

The ideas and business proposals fellows developed were examined not only by the faculties but also by the director of Stanford Biodesign. They examined the patent situation (14 IP submissions by six companies) and how innovative the medical technology they proposed was. After the faculties and the director agreed to the business proposals, the fellows were able to graduate the Biodesign course. This process is essential to make sure that their proposals are innovative and worth pursuing.

Out of 12, ten members are medical or dental doctors while two have engineering background. Each start-up has at least one doctor (CEO or non-CEO). Four companies have CEO of doctors. It is speculated that they have strong desire or mission mind that they would be able to help patients and medical professionals around the world by innovating medical technologies.

As shown in Table 7, some ventures had already secured VC investments, proving that the businesses were considered valuable and worth investing in, after considering certain risks from the viewpoint of third party VC. Although ongoing evaluation is needed, the results, the entrepreneur results of Japan Biodesign are recognized notable.

### Limitations

This report was based on assessment across four terms only, whereas more definite evaluation of the development of leadership talent and entrepreneurship as outcomes of education would require longer-term tracking. Continued surveys in the coming years would be most desirable in this regard. Moreover, the presumed lack of education programs for medical device innovation in Japan, prevented comparison with other programs. Similarly, we could not find academically comparable data from overseas organizations, except for Stanford University. By establishing future collaboration with other universities, both in Japan and overseas, the surveys could yield broader data from which meaningful comparisons can be drawn, which is highly desirable.

## Conclusion

Although it was suggested that team learning- and business-related educational aspects require improvement, the Japan Biodesign education initiative—with its self-devised externship program—shows definite positive results regarding medical device innovation. While the general situation in Japan reflects limited entrepreneurial awareness and a low new business entry rate, most fellows (95.7%) of Biodesign Japan, excluding company employees, wanted to start a new business. Since the program properly addresses general entrepreneurship factors, it objectively indicates the effectiveness of education in promoting entrepreneurship orientation and provides a platform for setting up a new business, followed by innovation.

With continued education on innovation and development of leadership talent, more human resources and talent will become available in the field of medical device innovation, which would result in creating an ecosystem of medical technologies and thus a favorable cycle of talent, innovation, industry, and ultimately, positive contribution to global healthcare.
